# Quality measures of two-stage newborn hearing screening: systematic review and meta-analysis

**DOI:** 10.3389/fpubh.2025.1566478

**Published:** 2025-04-16

**Authors:** Kirsi Manz, Uta Nennstiel, Carola Marzi, Ulrich Mansmann, Inken Brockow

**Affiliations:** ^1^Faculty of Medicine, Institute for Medical Information Processing, Biometry and Epidemiology (IBE), LMU Munich, Munich, Germany; ^2^Department of Hematology, Hemostasis, Oncology and Stem Cell Transplantation, Hannover Medical School (MHH), Hannover, Germany; ^3^Bavarian Health and Food Safety Authority, Oberschleissheim, Germany

**Keywords:** hearing screening, newborn, meta-analysis, referral rate, transient evoked otoacoustic emissions, automated auditory brainstem response

## Abstract

**Introduction:**

Newborn screening for hearing impairment (NHS) is a crucial public health issue worldwide. Often, a two-stage screening with two different testing approaches is used. We aimed to investigate the optimal screening algorithm, based on data from the literature published in the last 30 years. A particular focus of the study was to synthesize the existing evidence on two-stage newborn hearing screening regarding the refer rate (RFR), the percentage of children that did not pass the second test or were lost after the first test.

**Methods:**

We searched MEDLINE and Scopus for studies on two-stage NHS using transient evoked otoacoustic emissions (TEOAE) or automated auditory brainstem response (AABR). All studies on newborns who received their first test as an inpatient and a second test up to 1 month later were eligible. Random effects meta-analysis was performed to estimate RFR. Risk of bias was assessed using QUADAS-II. The unfunded study was registered in PROSPERO (CRD42023403091, available at https://www.crd.york.ac.uk/PROSPERO/view/CRD42023403091).

**Results:**

Eighty-five study protocols, including over 1,120,000 newborns, met the inclusion criteria. Certainty in the evidence was rated as moderate.

**Discussion:**

Strategies that did not involve changes to the screening method had a lower RFR. AABR-AABR: RFR = 1.3% [95% confidence interval (CI): 0.9, 1.8%], TEOAE-TEOAE: RFR = 2.7% (CI: 2.2, 3.2%), TEOAE-AABR: RFR = 3.9% (CI: 2.9, 5.1%), AABR-TEOAE: 5.9% (CI: 5.0, 6.9%). Consequently, where feasible, changing the screening method at the second screening should be avoided in order to minimize the number of follow-up examinations.

## 1 Introduction

A properly functioning auditory system is essential for a child’s acquisition of spoken language. Early intervention is critical for age-appropriate spoken language development in children with hearing impairment (1–5). Therefore, the Joint Committee on Infant Hearing strives for a start of interventions no later than at 3 months of age (6). Since the late 1990s, two screening methods have been available which allow a very early diagnosis: the measurement of transient evoked otoacoustic emissions (TEOAE) and automated auditory brainstem response (AABR) (7). The availability of these two methods, together with the high prevalence of the condition (1.3 per 1,000 newborns (8)), the existence of effective treatment options (e.g., hearing aids and cochlear implants), and the fact that early intervention has been shown to have positive health effects and a positive cost-benefit ratio (9–11), make congenital hearing impairment an appropriate target for inclusion in a screening program (12, 13). Universal newborn hearing screening (UNHS) was included in standard care in Germany in 2009, as specified by the Federal Joint Committee (G-BA) in §§ 47 to 57 of the Pediatrics Directive. The Pediatrics Directive outlines a two-step screening algorithm in well babies, beginning with initial TEOAE measurement in both ears, followed by bilateral AABR measurement if the initial test is not passed, and defines quality criteria. The utilization of distortion product otoacoustic emission (DPOAE) is not allowed as a screening method in this algorithm. DPOAE thresholds are limited to 50 dB HL, and the goal of newborn hearing screening (NHS) is to detect bilateral hearing loss with a threshold of ≥35 dB HL (14).

The effectiveness of any screening program depends on a number of factors, one of which is its specificity. With regard to the NHS program, the refer rate (RFR) is a relevant factor in this context, including newborns who are lost to follow-up after not passing the initial test, as well as those who receive a “fail” result on the second test. In practice, this equates to the percentage of screened infants who require referral to a pediatric audiologist for further diagnostic examinations after not passing the screening tests (positive screening).

It is crucial to strive for a minimal RFR, as it also encompasses false-positive findings as well as babies who are lost to follow-up. False-positive findings are of particular concern as they can cause anxiety for affected families and necessitate a costly and time-consuming intensive assessment, thereby further straining scarce resources in pediatric audiology practices and outpatient clinics.

The number of newborns lost to follow-up can be reduced by improving the practicality of the process, thereby increasing staff compliance. The occurrence of false-positive screening results, for example those resulting from amniotic fluid or debris in the auditory canal or a noisy environment, can be reduced by employing multi-step testing, with reasonable possible algorithms being TEOAE-TEOAE, AABR-AABR, or TEOAE-AABR.

The advantage of TEOAE is that it is a cost-effective and easily applicable method, whereas AABR is more accurate in terms of identifying false-positive results and is also capable of detecting brainstem hearing loss (6). The German Federal Joint Committee (G-BA) has established that a “fail” result of the initial TEOAE examination should be validated by an AABR in order to keep the RFR as low as possible. The quality target is an RFR that does not exceed 4% (14). The UK even requested lower RFRs (acceptable: 3%, achievable: 2%) (15).

Nevertheless, evaluations of the German NHS for the years 2011/12 (8) and 2017/18 (16, 17) have shown that the recommended screening-algorithm (TEOAE-AABR) is often not followed. In more than 50% of cases where infants do not pass the first test, a second TEOAE measurement is performed instead of the required AABR. Additionally, this second TEOAE test yielded a “fail” result in only about 10% of cases, compared to 20% for infants who underwent a second test using AABR. The failure rate of the second test was particularly high when the screening method was altered. Analysis of these data demonstrated that the second test showed the lowest failure rate with the TEOAE-TEOAE algorithm at 9.62%, while the highest rate was observed with TEOAE-AABR at 26.59%.

Accordingly, international recommendations suggest that a second TEOAE test should be performed after an initial TEOAE result of “fail” in newborns without risk factors for hearing impairment (“well-babies”) (6, 15). In light of these recommendations and the results of the NHS evaluation in Germany, it was necessary to evaluate whether the established algorithm in Germany (TEOAE-AABR) is a viable and optimal option, potentially applicable to other countries as well. Therefore, this study reviewed the current literature to investigate the quality of available screening tests and to find the optimal screening algorithm based on data published in the last 30 years. The screening algorithm should be cost-effective and easy to apply, with high sensitivity to detect all hearing losses, and high specificity to minimize the occurrence of false-positive results. The study focused specifically on synthesizing the existing evidence related to RFR in the two-stage NHS to determine the best two-step screening algorithm based on one of the two tests (TEOAE and AABR).

## 2 Methods

### 2.1 Model

The study population consists of newborns with relevant hearing impairment (“diseased,” D^+^) and those without (“healthy,” D^−^). The prevalence of hearing impairment in the population is unknown. The first stage of screening is performed using a method with an unknown sensitivity of SE1 and an unknown specificity of SP1. The observed test positivity rate of the first stage (PR1) defines the percentage of newborns who should receive a second test and are considered “*failed*.” Thus, the rate of positive results from the first stage is also referred to as the “failure rate.” However, not all newborns with a positive first test proceed to the second stage. The observed proportion *ρ* of positively tested newborns is *lost* (“loss rate”). This loss is assumed to be independent of hearing status (newborns drop out for reasons unrelated to the first test result). Thus, only a proportion of (1 − *ρ*) newborns undergo the second stage screening test.

The second stage of screening is performed using a method with an unknown sensitivity of SE2 and an unknown specificity of SP2. Again, the proportion of test-positives among the children in the second stage is of interest. The proportion of newborns who “fail” both tests, together with those who are lost after a first positive test, form the refer rate RFR. If the prevalence of hearing impairment is low, we obtain the following relationship connecting observed variables with an unknown parameter (SP2): RFR = PR1 * {*ρ* + (1 − *ρ*)*(1 − SP2)}. For small prevalence estimates, the RFR is linearly related to the failure (positive) rate of the first stage PR1. This linear relationship is determined by the loss rate *ρ* and the specificity of the second stage test SP2.

In the Supplementary Data 1 and Supplementary Figure 1, we provide a detailed mathematical description of how unobserved quantities of the second stage screening process relate to the observed quantities (unobserved: SE1, SP1, SE2, SP2, prevalence; observed PR1, PR2, *ρ*).

### 2.2 Study protocol

The meta-analysis was prospectively registered in PROSPERO (CRD42023403091). The amended review protocol can be found at and downloaded from https://www.crd.york.ac.uk/prospero/display_record.php?ID=CRD42023403091.

### 2.3 Eligibility criteria

For this review, the following PICO criteria were applied:

(P) Population: (Well) babies undergoing a two-stage hearing screening: (1) Initial screening as an inpatient in the maternity clinic; (2) Second test up to a maximum of 1 month later; (3) No use of the test method distortion product otoacoustic emissions (DPOAE); (4) Not exclusively newborns from NICU (neonatal intensive care unit).
(I) Intervention: Two-stage hearing screening using TEOAE, AABR, or a combination of both.
(C) Comparator: not applicable.
(O) Outcome: RFR after two screening steps.

The population should preferably consist of well babies. Studies that included newborns with risk factors for hearing impairment or babies from the NICU were included if the study data did not clearly distinguish between well babies and these newborns. However, studies that included only newborns with risk factors or from the NICU were excluded from this review.

The review considered studies in which both the first and the second tests were completed within 1 month after birth. The rationale behind this strict inclusion criteria was to ensure maximum homogeneity among the studies to ascertain that differences were attributable to the selected screening algorithm rather than to differences in study setting or patient age.

### 2.4 Search method

We searched MEDLINE using PubMed and Scopus for relevant articles without language or geographic restrictions from the time of their inception through February 9th, 2024. The following search strategy was used for both databases:

*(“newborn hearing screening”) OR (“neonatal hearing screening”) OR (“infant hearing screening”) OR (((“newborn screening”) OR (“neonatal screening”) OR (“infant screening”)) AND (“hearing”)) OR ((“hearing screening”) AND (“newborn” OR “neonatal” OR “infant”))*

A broader search strategy without specification of test method (TEOAE or AABR) was chosen to avoid missing relevant publications that may not specify the test method in the title or abstract. This decision was based on a small pilot search conducted to test the search strategy, which showed that including the type of screening test in the search resulted in missing some articles that were already known to be relevant to the review.

All articles had an abstract in English. If the full texts of the selected articles were not written in a language that the authors speak, online translation tools (DeepL, Google Translator) were used to translate them into English where possible. Only peer-reviewed publications were included.

### 2.5 Study records

The search strategy was saved in Citavi version 6.11. The data from the selected publications were extracted into Excel spreadsheets.

One reviewer searched the information sources and screened the titles and abstracts of the identified studies for inclusion and classified each study as eligible or ineligible. The study was classified as potentially eligible if it could not be clearly excluded based on its title and abstract. The full texts of all (potentially) eligible studies were then retrieved and reviewed by two additional reviewers. Again, studies were marked as eligible or ineligible for inclusion and the selection was discussed with the first reviewer until all three reviewers agreed. The first reviewer extracted the required data and made the preliminary decision on study inclusion based on the availability of the data for extraction. The second reviewer double-checked the extracted data and made corrections, which were discussed with all three reviewers and led to the final inclusion of all studies with available data.

### 2.6 Data extraction and items

Data extracted for the study description included the following: first author and year of publication, screening test combination (i.e., TEOAE-TEOAE, TEOAE-AABR, AABR-TEOAE, AABR-AABR), years of screening, country, number of newborns screened, name of screening device(s), time of first test and second test, and whether the study was conducted only in well babies or also in newborns with risk factors/from the NICU.

Data extracted for quantitative analysis included the following: number of newborns screened with the first test and type of first test (TEOAE/AABR), number of newborns who passed the first test, number of newborns who did not pass the first test, number of newborns who did not pass the first test and did not return for the second test (lost-to-follow-up), number of newborns screened for the second time and type of second test (TEOAE/AABR), number of newborns passing the second test, number of newborns who did not pass the second test, and number of newborns referred after not passing both screening tests.

Studies providing data on simultaneous TEOAE and AABR testing were added to both screening test combinations: TEOAE followed by AABR and AABR followed by TEOAE. Otherwise, such studies would have had to be excluded as the order of the tests was not sequential.

The following data were derived from the variables collected: the failure rate after the first test step was calculated as the quotient of the number of newborns who did not pass the first test and all newborns screened in the first step. Similarly, the failure rate after the second test step was calculated as the quotient of the number of newborns who did not pass the second test and all newborns screened in the second step. The RFR was calculated as the sum of the number of newborns who did not pass the second test and the number of newborns who did not pass the first test and did not return for the second test, divided by the total number of newborns screened.

### 2.7 Outcomes and prioritization

Data were sought to calculate the RFR after the two-step NHS, with the failure rate after the first test included in the analysis. For the analysis, it was essential to obtain the number of infants who passed and did not pass each screening step. Therefore, studies that only reported the results after both screening steps could not be included in the review. This review focused mainly on well babies, but studies conducted in both well babies and newborns with risk factors or from the NICU were also included.

### 2.8 Risk of bias in individual studies and publication bias

The quality of the included studies was assessed using a modified version of the QUADAS-II tool developed for diagnostic accuracy studies (18). Of the original four domains, the “reference test” domain was not applicable to our study because we focused on the two-stage screening process without knowing the true hearing loss status. Therefore, we replaced the domain “index test” by “first test” and “reference test” by “second test.” The risk of bias for each included study was assessed independently by two reviewers. Disagreements were resolved by discussion with a mediator. The risk of bias was assessed at the study level.

Publication bias was not expected to have a significant impact on the literature found. It was assumed that all studies on two-stage NHS would be worthy of publication, as they describe not only the quality of the NHS, but also its implementation and problems. Selective reporting within studies, e.g., favoring one of the two screening tests, is also unlikely to be a problem. Therefore, methods to assess the risk of publication bias were not used in this meta-analysis.

### 2.9 Confidence in cumulative evidence

The strength of the overall body of evidence was assessed using Grading of Recommendations, Assessment, Development and Evaluation (19).

### 2.10 Subgroup analyses

Subgroup analyses were not specified in the study protocol. However, based on the characteristics of the included studies, we decided to analyze only well-baby studies as a subgroup. An additional *post hoc* sensitivity analysis was performed to assess the effect of outliers in the TEOAE-TEOAE group.

### 2.11 Statistical analysis

Continuous variables were summarized by median (minimum–maximum) or presented as box plots, and categorical variables were presented by frequency (%). Due to the exploratory nature of our study, adjustment for multiple testing was not considered. Statistical significance was claimed at 5% level (*p* < 0.05) or for non-overlapping 95% confidence intervals. Calculations were performed using R Version 4.3.2 (20). Random effects meta-analysis of the RFR was performed using the R package *rmeta* and the DerSimonian–Laird approach. Heterogeneity indices *Q* and *I*^2^ were calculated using the random effect estimates and random effect weights. Meta-regression was performed using the *rma* function of the package *metafor*. For the visualization of the risk of bias, we used the source code of the *rob_summary* function of the *robvis* package to generate a similar graph adapted to our needs. All data and analysis scripts are available in the Open Science Framework repository at https://osf.io/nuk4p/.

## 3 Results

**Figure 1:**
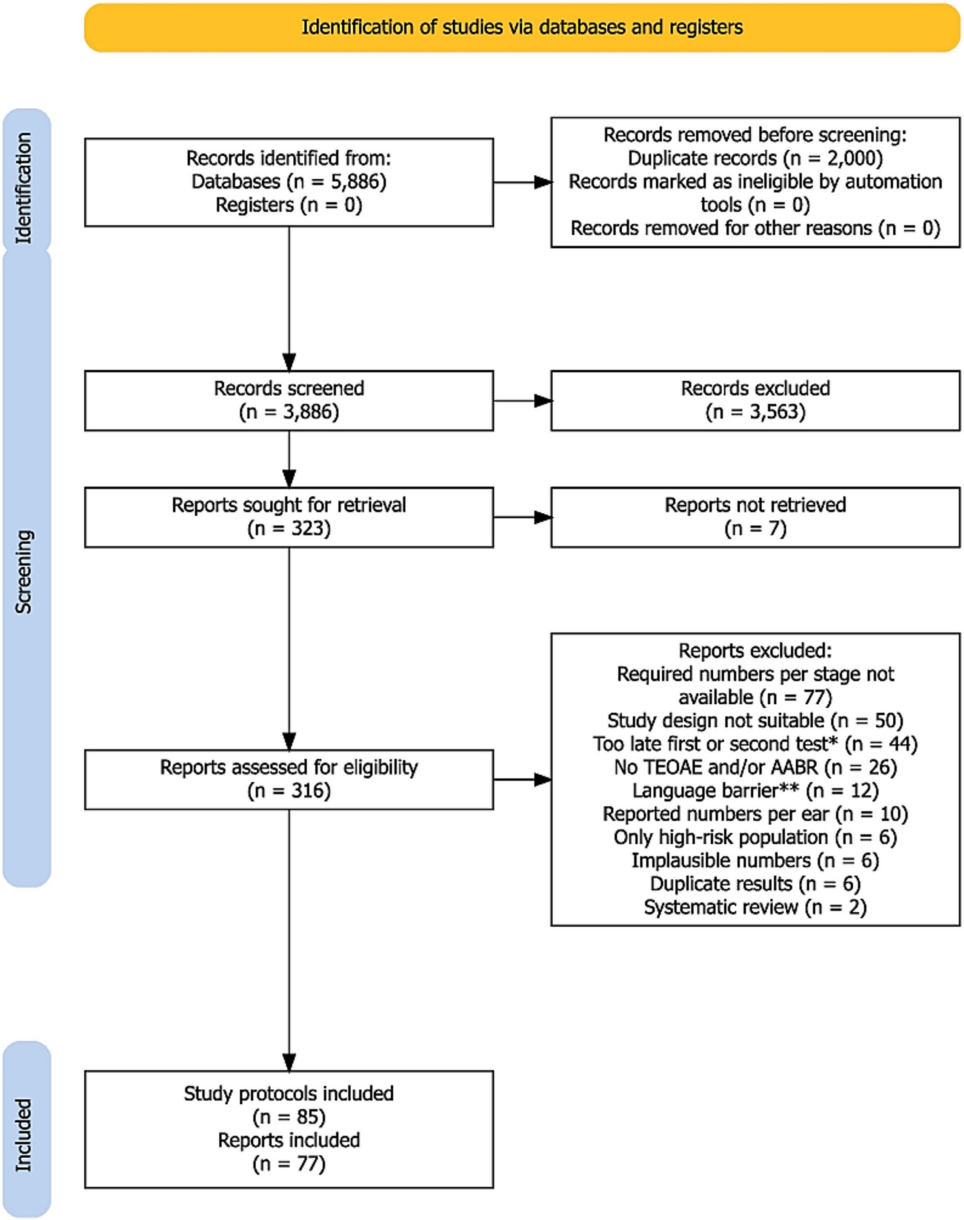
PRISMA flow diagram of the search and study selection process. ^*^Includes also studies where the time point of the first or second test was not specified. ^**^Language barrier refers to reports that could not be automatically translated into English online. A detailed list of the 239 excluded reports is available from the authors upon request.

The PRISMA flow diagram for the search and study selection process is shown in Figure 1. Out of the 5,886 records identified (PubMed: *n* = 3,356, Scopus: *n* = 2,530), 2,000 duplicates were removed prior to screening. From a total of 3,886 records screened, a total of 3,563 records were excluded because the titles and abstracts of these articles were not relevant to our research question. A full-text search was conducted on the remaining 323 records. Seven records could not be retrieved. Out of the 316 reports that were screened, 239 were excluded. In many cases this was due to a failure to provide the required data at each screening step (*n* = 77), an unsuitable study design (*n* = 50), or not performing the first or second test within the specified time frame of 1 month (*n* = 44). Additional reasons for exclusion are listed in Figure 1. *N* = 7 reports comprised more than one screening protocol [*n* = 6 two protocols (21–26) and *n* = 1 three protocols (27)]. Of the reports with more than one protocol, three (24, 26, 27) provided data on simultaneous TEOAE and AABR testing and were included in both the TEOAE-AABR and AABR-TEOAE groups. A total of 77 reports with 85 study protocols were included in the meta-analysis.

The analysis included 85 study protocols from 77 reports (7 of those with more than one study protocol) with 1,125,617 newborns. Table 1 provides an overview of the study characteristics.

**Table 1:** Characteristics of the included study protocols.

Screening protocol	Author	Year(s)	Country	No. of screened newborns	Time of first test	Time of second test	High-risk/NICU included^a^
TEOAE-TEOAE	Aidan et al. (35)	1995–1997	France	1,421	48 h	Within the first month	No
TEOAE-TEOAE	Alanazi (36)	2020^d^^,^^j^	Saudi Arabia	20,171	Before hospital discharge	Within 2 weeks	Yes
TEOAE-TEOAE	Arjmandi et al. (37)	2009–2010	Iran	1,232	10 days^e^	After 2–3 weeks	Yes
TEOAE-TEOAE	Arora et al. (38)	2017–2019	India	1,200	<72 h	After 3–4 weeks	Yes
TEOAE-TEOAE	Arslan et al. (39)	2007–2008	Türkiye	2,229	Within 7 days, before hospital discharge	Within 15 days after 1st screening	Yes
TEOAE-TEOAE	Azizi et al. (40)	2006–2007	Iran	3,818	<48 h	After 2–4 weeks	Yes
TEOAE-TEOAE	Benito-Orejas et al. (21)	2001–2003	Spain	2,454	Within 48 h, NICU: infants tested on discharge day	Within the first month	Yes
TEOAE-TEOAE	Bevilacqua et al. (41)	2010^i^^,^^j^	Brazil	11,466	24 h	Within 20 days after birth	Yes
TEOAE-TEOAE	Busse et al. (22)	2018–2019	Albania	778	24–48 h	After 14 days	No
TEOAE-TEOAE	Calevo et al. (42)	2001	Italy	3,238	48–72 h	Within the third week of life	Yes
TEOAE-TEOAE	Calevo et al. (43)	2002–2004	Italy	32,502	48–72 h	Within the third week of life	No
TEOAE-TEOAE	Cavalcanti et al. (44)	2007–2009	Brazil	3,724	36–48 h	1 week after first test	No^f^
TEOAE-TEOAE	Chapchap and Segre (45)	1996–1999	Brazil	4,196	48–72 h (NICU: prior discharge)	Within 30 days (no specification)	Yes
TEOAE-TEOAE	Clarke et al. (23)	2001–2002	United Kingdom	81	21 h^b^	Before hospital discharge	No
TEOAE-TEOAE	De Capua et al. (46)	1998–2006	Italy	19,700	96 h^b^, NICU: at post-menstrual age of 37–41 weeks	10–20 days after birth	Yes
TEOAE-TEOAE	Diego Gimenes Lopes et al. (47)	2016–2019	Brazil	1,553	Before hospital discharge	Within 30 days	Yes
TEOAE-TEOAE	Eibenstein et al. (48)	2007–2012	Italy	4,579	Before hospital discharge	Within 2 weeks	No
TEOAE-TEOAE	Eibenstein et al. (49)	2013–2014	Italy	3,120	Before hospital discharge	Within 2 weeks	No
TEOAE-TEOAE	Erturk et al. (25)	2002–2003	Türkiye	500	Before hospital discharge	After 3 weeks	Yes
TEOAE-TEOAE	Escobar-Ipuz et al. (50)	2007–2017	Spain	9,350	<48 h	Before hospital discharge	Yes
TEOAE-TEOAE	Farahani et al. (51)	2013	Iran	2,784	Day 1 or day 2	1–2 weeks after first test	No
TEOAE-TEOAE	Ferlito et al. (52)	2018	Italy	37,562	48–72 h	Within the first month	Yes
TEOAE-TEOAE	George et al. (53)	2015	Bahrain	1,834	Before hospital discharge	1 week after discharge	Yes
TEOAE-TEOAE	Fusetti et al. (54)	2005–2007	Italy	1,400	Within 1 week of life	2 weeks after first test	No
TEOAE-TEOAE	Gilbey et al. (55)	2010–2011	Israel	4,958	Before hospital discharge	Day after 1st screening	No
TEOAE-TEOAE	Guastini et al. (56)	2006–2009	Italy	8,671	48–72 h, NICU: when stable general condition	2 weeks after first test	Yes
TEOAE-TEOAE	Gül et al. (28)	2010–2011	Türkiye	2,363	Before hospital discharge	2 weeks after first test	Yes
TEOAE-TEOAE	Habib and Abdelgaffar (57)	1996–2004	Saudi Arabia	11,986	<48 h	5th day	No
TEOAE-TEOAE	Hatzopoulus et al. (58)	2003–2004	Albania	450	24–48 h	Within 4 weeks of first test	No
TEOAE-TEOAE	Jakubikova et al. (59)	2003^j^	Slovak Republic	3,048	4th–12th day at hospital discharge	1 month after first test	Yes
TEOAE-TEOAE	Kayiran et al. (60)	2004–2009	Türkiye	8,052	Before hospital discharge	1 week later	No
TEOAE-TEOAE	Konukseven et al. (61)	2007–2009	Türkiye	1,917	<48 h	After 10 days	No
TEOAE-TEOAE	Korres et al. (62)	2006^g^^,^^j^	Greece	22,195	48–72 h	After 1 month	Yes
TEOAE-TEOAE	Korres et al. (63)	2006^h^^,^^j^	Greece	25,032	48–72 h	After 1 month	Yes
TEOAE-TEOAE	Kosmidou et al. (64)	2018–2020	Greece	1,491	First days of life	Within the first month	No
TEOAE-TEOAE	Lin et al. (65)	2000–2002	Taiwan	5,938	>24, before discharge	1 month later	No
TEOAE-TEOAE	Lotfi and Movallali (66)	2002–2004	Iran	7,718	3–36 h	15–30 days old	No
TEOAE-TEOAE	Magnani et al. (67)	2010–2013	Italy	10,359	24–48 h	Within 3 weeks from birth	No
TEOAE-TEOAE	Molini et al. (68)	2010–2012	Italy	18,796	24–36 h	1 month of age	No
TEOAE-TEOAE	Molteni (69)	1999–2005	Italy	10,454	3rd day of life	1 month	Yes
TEOAE-TEOAE	Pastorino et al. (70)	1997–2001	Italy	19,290	36–48 h (vaginal delivery), 3–5 days (C-section)	15–30 days after discharge	No
TEOAE-TEOAE	Pedersen et al. 2008 (71)	2006	Denmark	1,627	2–30 days	2–30 days	Yes
TEOAE-TEOAE	Prpic et al. (72)	2002–2006	Croatia	11,746	2 or 3 days (NICU: when stable)	3 weeks after first test	Yes
TEOAE-TEOAE	Pyarali et al. (73)	2021	Pakistan	267	Before hospital discharge	Before hospital discharge	Yes
TEOAE-TEOAE	Satish et al. (74)	2015–2017	India	26,487	<48 h	1 week after first test	Yes
TEOAE-TEOAE	Sennaroglu and Akmese (75)	2009–2010	Türkiye	1,840	Before hospital discharge/within 10 days	After 15 days	Yes
TEOAE-TEOAE	Sequi Canet et al. (76)	2002–2013	Spain	14,015	As late as possible prior discharge	Age of 1 month	Yes
TEOAE-TEOAE	Sheng et al. (27)	2018–2019	China	1,340	<48 h	<48 h	No
TEOAE-TEOAE	Tasci et al. (77)	2007–2008	Türkiye	15,323	24–48 h	Within 10 days	No
TEOAE-TEOAE	Tatli et al. (78)	2002–2003	Türkiye	711	Last working day prior hospital discharge	1 week after first test	Yes
TEOAE-TEOAE	Unlu et al. (79)	2009–2013	Türkiye	2,933	Day 5	Day 15	No
TEOAE-TEOAE	Vaid et al. (80)	2005–2007	India	1,238	<72 h	1 month	No
TEOAE-TEOAE	Vos et al. 2014 (81)	2007–2012	Belgium	245,219	48–72 h	Day 3 or 4 (following day after first test)	No
TEOAE-TEOAE	Welzl-Müller et al. (82)	1997^j^	Austria	2,338	Within the first days	Within 1–2 days after first test	No
TEOAE-TEOAE	Yorulmaz et al. (29)	2011–2016	Türkiye	13,693	Before hospital discharge	14 days later	Yes
TEOAE-AABR	Dort et al. (24)	2000^j^	Canada	64	Before hospital discharge	Before hospital discharge	Yes
TEOAE-AABR	Lin et al. (83)	2004–2005	Taiwan	3,540	>48 h	Before hospital discharge	Yes
TEOAE-AABR	Mazlan et al. (84)	2010–2019	Malaysia	50,633	<24 h	Within 2 weeks	Yes
TEOAE-AABR	Nennstiel-Ratzel et al. (85)	2003–2005	Germany	16,767	Before hospital discharge	Before hospital discharge	Yes
TEOAE-AABR	Olusanya et al. (86)	2005–2006	Nigeria	1,150	24–48 h	Before hospital discharge	No
TEOAE-AABR	Olusanya and Bamigboye (87)	2005–2007	Nigeria	4,718	24 h, SCBU^c^: shortly before discharge	Before hospital discharge	Yes
TEOAE-AABR	Ong et al. (26)	2018	Philippines	247	Before hospital discharge	Before hospital discharge	Yes
TEOAE-AABR	Pasha et al. (88)	2006–2014	Iran	40,930	Before hospital discharge	1 month of age	No
TEOAE-AABR	Sheng et al. (27)	2018–2019	China	2,005	<72 h	<72 h	No
AABR-TEOAE	Dort et al. (24)	2000^k^	Canada	64	Before hospital discharge	Before hospital discharge	Yes
AABR-TEOAE	Ong et al. (26)	2018	Philippines	247	Before hospital discharge	Before hospital discharge	Yes
AABR-TEOAE	Sheng et al. (27)	2018–2019	China	2,005	<72 h	<72 h	No
AABR-AABR	Alothman et al. (89)	2021	Saudi Arabia	199,034	24 h/prior discharge	Prior discharge	Yes
AABR-AABR	Al Shamisi and Roy (90)	2010–2019	United Arab Emirates	37,661	Before hospital discharge	1 month	Yes
AABR-AABR	Ayas and Yaseen (91)	2017–2020	United Arab Emirates	1,821	24–48 h or shortly before discharge	2 weeks after first test	No
AABR-AABR	Benito-Orejas et al. (21)	2004–2006	Spain	3,117	Within 48 h, NICU: tested on discharge day	Within 1 month after birth	Yes
AABR-AABR	Busse et al. (22)	2018–2019	Albania	1,129	24–48 h	After 14 days	Yes
AABR-AABR	Clarke et al. (23)	2001–2002	United Kingdom	81	24 h^b^	Before hospital discharge	No
AABR-AABR	Clemens and Davis (92)	1999–2000	United States	3,142	Before hospital discharge	Within 12–24 h after first test, prior discharge	No
AABR-AABR	Erturk et al. (25)	2002–2003	Türkiye	500	Before hospital discharge	After 3 weeks	Yes
AABR-AABR	Fan et al. (93)	2005–2008	Taiwan	7,139	Before hospital discharge	At 1 month of age	No
AABR-AABR	Gupta et al. (94)	2011–2012	India	2,265	24–48 h, preterm babies >34 postmenstrual weeks	Within 7 days after 1st test	Yes
AABR-AABR	Huang et al. (95)	2009–2010	Taiwan	15,790	24–36 h	36–60 h of age	Yes
AABR-AABR	Iwasaki et al. (96)	2000–2001	Japan	4,085	48–72 h	5–6 days after birth, prior discharge	Yes
AABR-AABR	Kelly et al. (97)	2014–2016	United States	31,984	6 h (vaginal delivery), 12 h (C-section)	Before hospital discharge	No
AABR-AABR	Messner et al. (98)	1998–1999	United States	5,771	<24 h	Before hospital discharge	No
AABR-AABR	Oruc et al. (99)	2018	Türkiye	5,399	Before hospital discharge	15 days later	Yes
AABR-AABR	Shim et al. (100)	2005–2015	South Korea	3,059	24 h	Within 1 month	Yes
AABR-AABR	Tanyeri Toker et al. (101)	2020–2021	Türkiye	570	<72 h	7–15 days of age	No
AABR-AABR	Tsuchiya et al. (102)	1999–2004	Japan	8,979	Day 4	1 month of age	No

All studies are considered to be level of evidence 2b, individual cohort study/low-quality randomized control study.

a Studies that include only healthy newborns or exclude newborns with risk factors were scored as “no,” NICU, newborn intensive care unit.

b Median age at first test.

c SCBU, special care baby unit.

d Data from 3 years were analyzed.

e Mean age at first test.

f Originally only well babies, newborns transferred to special care baby unit were counted as losses to follow-up.

g Three years after program initiation.

h Additional data from two additional years were presented.

i During 3 years.

j Unknown dates, the publication year of the article is given.

All included studies are cohort studies and have a level of evidence 2b, individual cohort study/low-quality randomized control study, following definitions given in https://guides.library.stonybrook.edu/evidence-based-medicine/levels_of_evidence.

Of the 85 study protocols, *n* = 55 (64.7%) studies examined the TEOAE-TEOAE test combination, *n* = 9 (10.6%) examined the TEOAE-AABR test combination *n* = 3 (3.5%) examined the AABR-TEOAE test combination, and *n* = 18 (21.2%) examined the AABR-AABR test combination. The median study size across all study protocols was *n* = 3,238 newborns (min–max: 64–245,219). The median study size for TEOAE-TEOAE was *n* = 3,724 newborns (min–max: 81–245,219), for TEOAE-AABR it was *n* = 3,540 newborns (min–max: 64–50,633), for AABR-TEOAE it was *n* = 247 newborns (min–max: 64–2,005), and for AABR-AABR it was *n* = 3614 newborns (min–max: 81–199,034).

**Figure 2:**
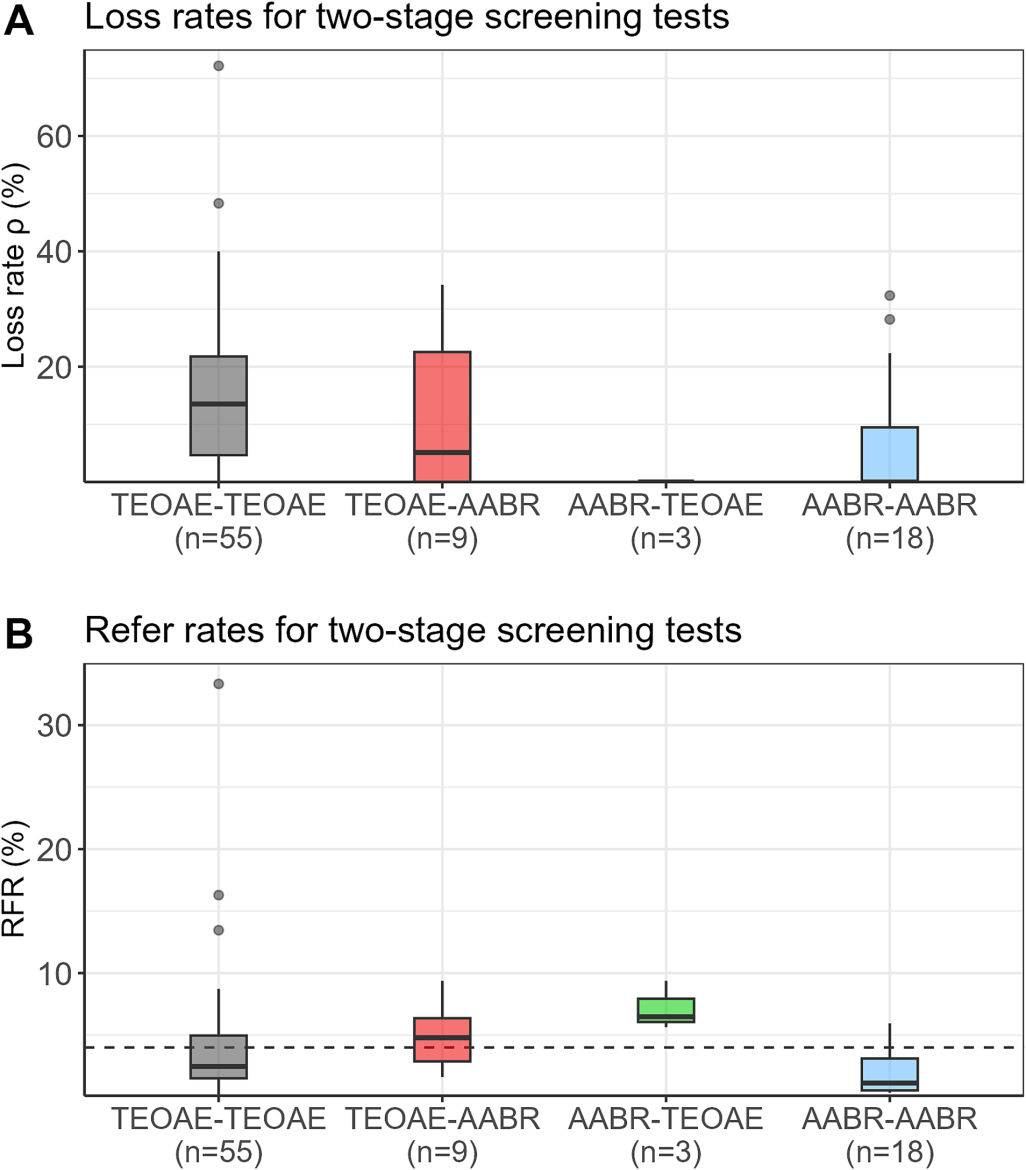
Loss rates (*ρ*) **(A)** and refer rates (RFR) **(B)** for the four different screening test combinations. The dashed horizontal line indicates the 4% threshold quality criteria defined in the Pediatrics Directive for the RFR. AABR, automated auditory brainstem response; TEOAE, transient evoked otoacoustic emission.

Figure 2A presents the loss rates of newborns who did not pass the first test and did not attend the second test for all test combinations. Depending on the study, loss rates of up to 72% were reported. Median loss rates were 14% for TEOAE-TEOAE, 5% for TEOAE-AABR, and zero for AABR-TEOAE and AABR-AABR. Figure 2B shows the RFR of all study protocols. Median RFRs of 2.5, 4.8, 6.5, and 1.1% were found for the TEOAE-TEOAE, TEOAE-AABR, AABR-TEOAE, and AABR-AABR combinations, respectively. All but three studies (23, 28, 29), reported RFR below 10%. Out of the 85 study protocols, 57 (67.1%) showed a RFR below 4%.

### 3.1 Random effects meta-analysis of RFR

**Figure 3:**
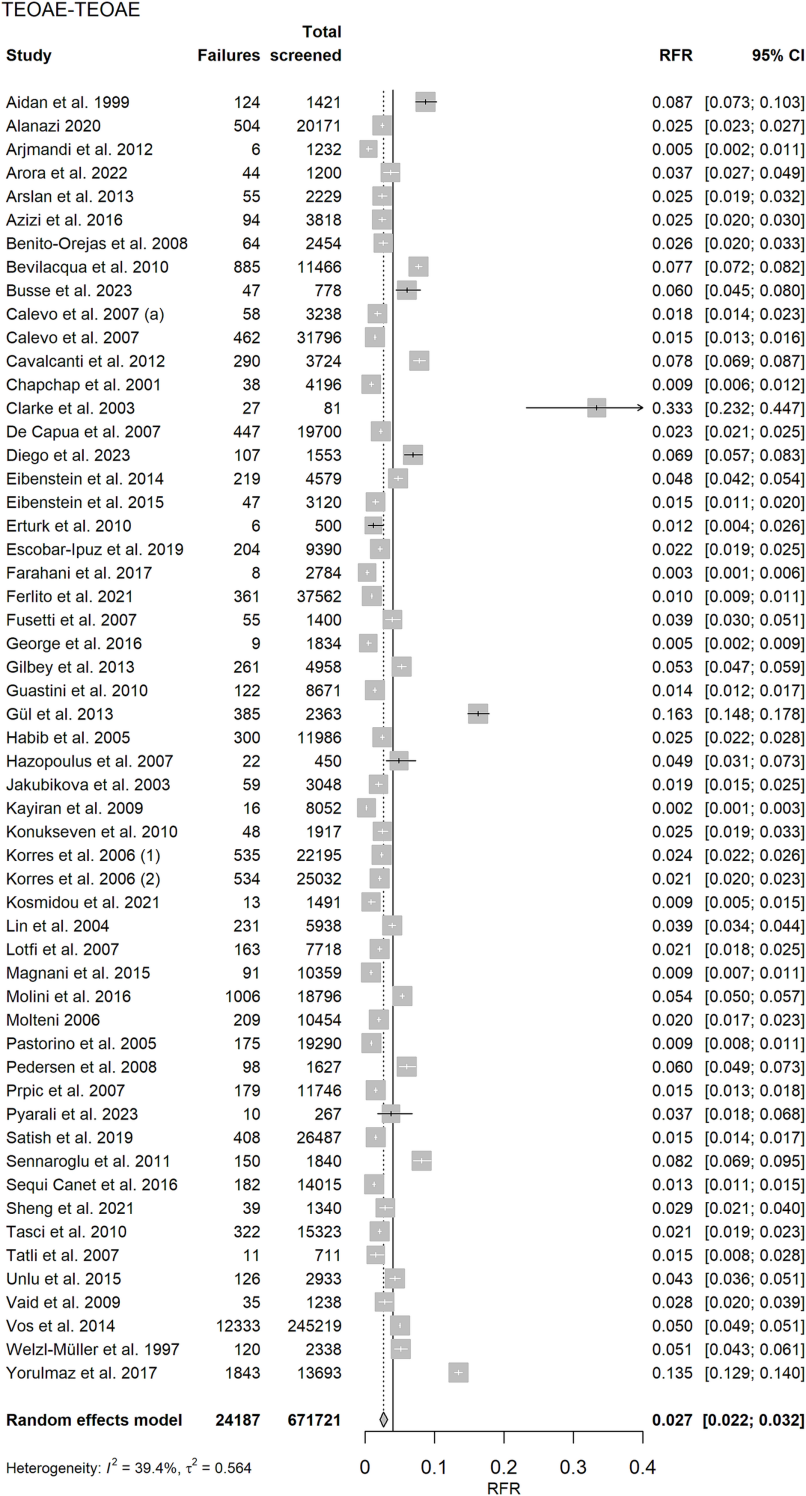
Random effects meta-analysis of refer rate (RFR) for the 55 TEOAE-TEOAE study protocols. The table shows the number of newborns who did not pass the first and second test (“Failures”), the total number of screened newborns (“Total screened”), and the RFR with 95% confidence interval (95% CI) for each study. The summary estimate, including the 95% CI is shown as a grey diamond on a scale ranging from 0 to 40%. The vertical solid line indicates the 4% threshold quality criteria defined in the Pediatrics Directive for the RFR. TEOAE, transient evoked otoacoustic emission.

**Figure 4:**
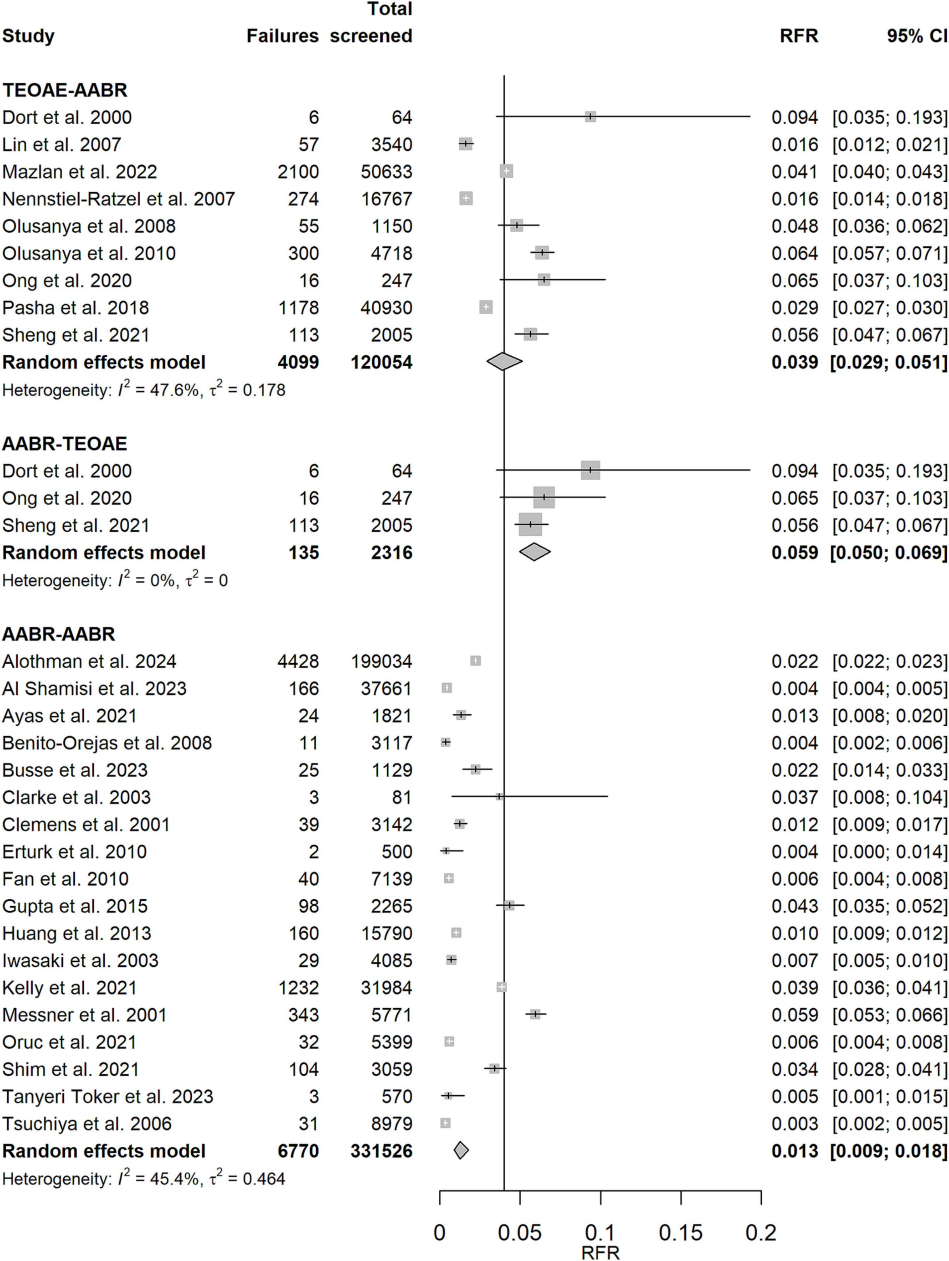
Random effects meta-analysis of refer rate (RFR) for TEOAE-AABR, AABR-TEOAE, and AABR-AABR study protocols. The table shows the number of newborns who did not pass the first and second test (“Failures”), the total number of screened newborns (“Total screened”), and the RFR with 95% confidence interval (95% CI) for each study. The summary estimate per test combination, including the 95% CI, is shown as a grey diamond on a scale ranging from 0 to 20%. The vertical solid line indicates the 4% threshold quality criteria defined in the Pediatrics Directive for the RFR. AABR, automated auditory brainstem response; TEOAE, transient evoked otoacoustic emission.

Forest plots of the random effects meta-analysis of RFR are presented in Figure 3 for the TEOAE-TEOAE test combination and in Figure 4 for the other test combinations. A summary of the meta-analysis results is presented in Table 2.

**Table 2:** Results of the random effects meta-analysis of the refer rate.

Test combination	RFR (95% CI)	*I* ^2^	tau^2^	*Q*	df	*p*
TEOAE-TEOAE	2.7% (2.2, 3.2%)	39.4%	0.564	89.14	54	**0.002**
TEOAE-AABR	3.9% (2.9, 5.1%)	47.6%	0.178	15.27	8	0.054
AABR-TEOAE	5.9% (5.0, 6.9%)	0%	0	1.76	2	0.416
AABR-AABR	1.3% (0.9, 1.8%)	45.4%	0.464	31.14	17	**0.019**

RFR, refer rate; CI, confidence interval; TEOAE, transient evoked otoacoustic emission; AABR, automated auditory brainstem response. The *p*-value shown in the right column relates to the Cochrane’s *Q* statistic (*Q*) for heterogeneity. Statistically significant *p*-values (*p* < 0.05) are printed in bold.

Strategies that do not involve a change in the screening test method showed the lowest RFR [AABR-AABR: RFR = 1.3% (CI: 0.9, 1.8%), TEOAE-TEOAE: RFR = 2.7% (CI: 2.2, 3.2%)]. The upper limits of their 95% confidence intervals are below the recommended quality threshold of 4%. When the screening test method is changed between stage 1 and stage 2, the corresponding 95% confidence intervals cover or exceed the 4% threshold [TEOAE-AABR: RFR = 3.9% (CI: 2.9, 5.1%), AABR-TEOAE: 5.9% (CI: 5.0, 6.9%)]. Studies of both screening combinations TEOAE-TEOAE and AABR-AABR show a moderate degree of heterogeneity as quantified by *I*^2^ (39.4 and 45.4%, respectively).

Excluding the TEOAE-TEOAE study protocol with a remarkably high RFR of 33.3% (23) and the two other studies with RFR >10% (28, 29), as shown in Supplementary Figure 2, reduces the summary estimate for RFR from 2.7% (CI: 2.2, 3.2%) to 2.4% (CI: 2.0, 2.8%). The results for RFR for well babies (Supplementary Figures 3, 4) are comparable to those of all studies: AABR-AABR and TEOAE-TEOAE show the lowest RFR (less than 4%). Changing the test method results in a higher RFR.

The results of the meta-regression of the failure rate of the first test and the RFR are presented in Supplementary Table 1 and Supplementary Figure 5. The results show that AABR-AABR is the best screening protocol.

### 3.2 Bias assessment

**Figure 5:**
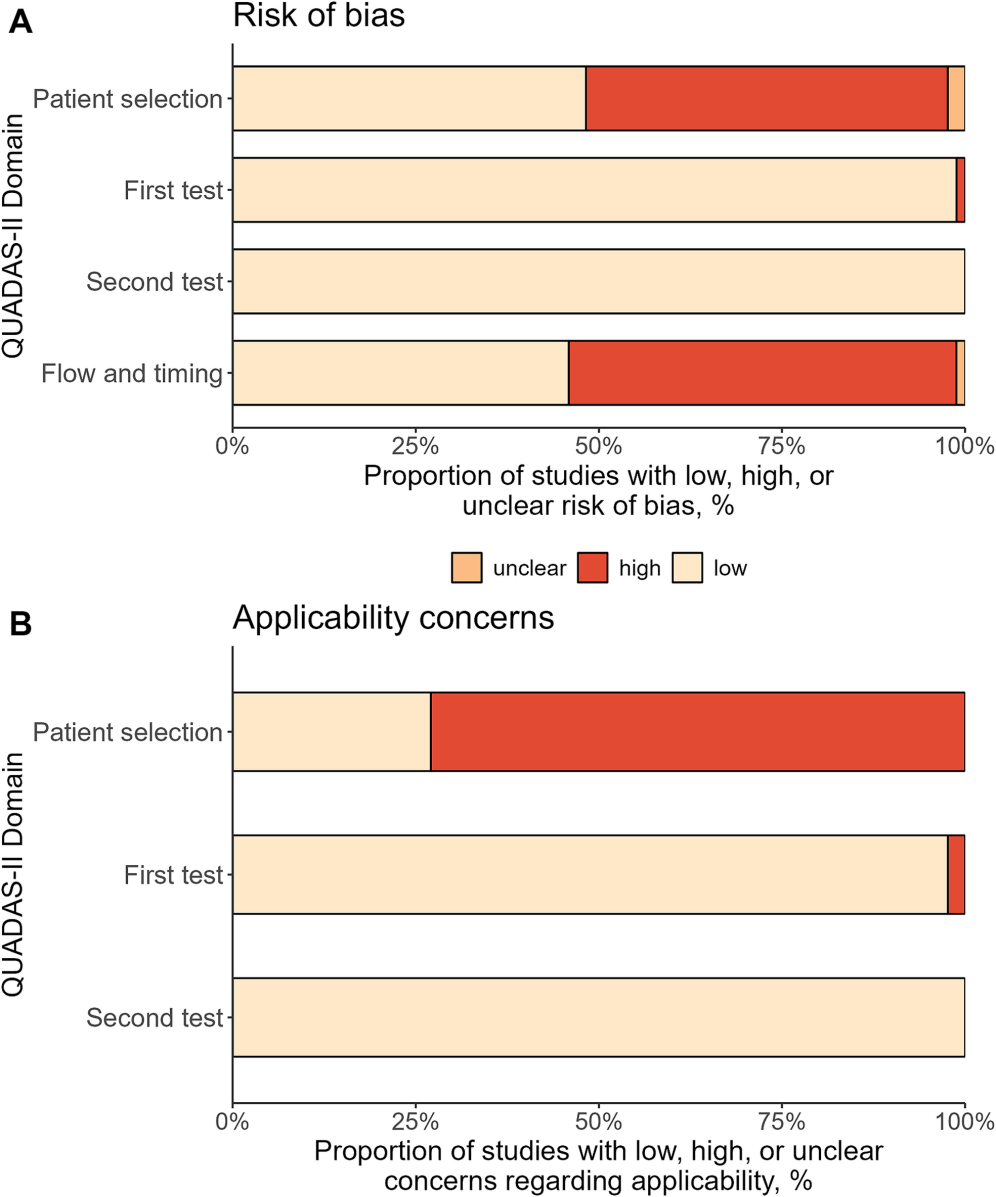
Bias assessment using QUADAS-II tool. The figure shows the percentage of the included 85 study protocols with low, high, or unclear risk of bias **(A)** and concerns regarding applicability **(B)**.

Figure 5 shows the results of the bias assessment. While there was a low risk of bias in the use of the tests (first test: 98.8%, second test: 100%), about half of the studies had a high risk of bias in patient selection and in flow and timing (49.4 and 52.9%, respectively). In terms of applicability concerns, about 3 out of 4 studies had high concerns for patient selection (72.9%). Similar to the risk of bias, both tests showed only low applicability concerns (first test: 97.6%, second test: 100%). The study-level bias assessment for all domains can be found in Supplementary Table 2.

The GRADE summary of findings table is included in Supplementary Table 3. We rated all evidence generated in this meta-analysis as moderate, which is the second highest GRADE evidence category. The level of evidence for the RFR from the random effects meta-analysis was lowered to moderate because of the moderate heterogeneity found, and for test combinations with test method change between the two stages because of the small number of pooled studies.

## 4 Discussion

A meta-analysis was conducted to identify the optimal screening algorithm for a two-stage NHS using combinations of TEOAE or AABR tests. The study analyzed 85 study protocols with over 1,120,000 newborns who completed both the first and second hearing tests within 1 month after birth. The results showed that the refer rate (RFR) was lower when there was no change in the screening method used. The aggregated RFR was 1.3% for the AABR-AABR test combination and 2.7% for TEOAE-TEOAE.

The following discussion focuses on newborn hearing screening in the German setting. However, we believe that the discussion will be of broader interest to other countries, as a two-stage algorithm is typically used in NHS programs. In all population-based health programs globally, regular monitoring, evaluation and quality management are essential to ensure the effectiveness, efficiency and sustainability of the program, thereby enabling continuous improvement. In the case of the NHS, this includes the validation of screening algorithms that should meet the required quality standards, particularly with respect to false-positive findings, while simultaneously ensuring cost-effectiveness and applicability in the clinical setting.

The RFR is an important quality parameter in the NHS, as screenings with a refer result must be followed up by a pediatric audiologist. These specialists are scarce in Germany (as well as in other countries), which can lead to long waiting times for families with children who have not passed the screening tests. In addition, false-positive results can cause unnecessary anxiety for parents (30). A low RFR in the NHS can be achieved primarily through a multi-step screening algorithm. Therefore, when the NHS was introduced in 2009, the German Pediatrics Directive required an AABR control if the first hearing test was not passed and an RFR of less than 4% at discharge, in line with national and international quality targets at the time (14, 31).

The German NHS evaluation data for the years 2011/2012 and the follow-up evaluation data for 2017/2018 both indicate that a second hearing test following an initial screening test with a “fail” result considerably reduces the RFR, as this second test was passed in over 80% (8, 16). However, it is worth noting, that this second measurement was performed with a TEOAE in more than half of the tests, which is contrary to the German Pediatrics Directive. In follow-up evaluation interviews this was explained by the longer measurement duration and the increased susceptibility to interference of the AABR. The follow-up evaluation analysis revealed a considerably higher rate of refer results in the second test when a different test method was used than in the first test (8, 16). This is in line with the finding of this meta-analysis that the highest RFR were observed when the test method was changed (TEOAE-AABR or AABR-TEOAE). The rational behind this observation remains unclear, but it may be attributable to the examiner’s minor familiarity with the less frequently used second stage screening method.

In the German follow-up evaluation, the TEOAE-TEOAE algorithm had a lower RFR (9.62%) than AABR-AABR (13.98%) (16). In contrast, in this meta-analysis the AABR-AABR test combination had the lowest RFR, even lower than that of the TEOAE-TEOAE algorithm. This was also observed in the subgroup analysis on “well babies” and may be attributed to the higher lost-to-follow-up rate in the studies reporting TEOAE-TEOAE results (see Figure 2). Likewise, the questionnaire-based EUSCREEN study demonstrated a lower RFR for programs utilizing aABR in comparison to those employing OAE exclusively (32). In addition, studies utilizing this test combination exhibited a significantly higher heterogeneity (see Tables 2, *Q* statistic) and were often based on routine clinical data, whereas data for the AABR-AABR test sequence were mainly derived from clinical studies. The AABR diagnostic is the “gold standard” for detecting most hearing disorders. However, since TEOAE offers the most practical screening setting and TEOAE-TEOAE has the second best refer rate, the authors recommend this combination for well-babies. This recommendation is also applicable on a global scale, particularly in developing countries, as TEOAE is a cost-effective and easily applicable method that does not necessitate the use of costly consumable materials. One limitation of TEOAE is its inability to detect retrocochlear causes of hearing loss, such as auditory neuropathy (AN). However, this condition is rare in well babies as, i.e., Boudewyns et al. (33) estimated the incidence of AN in a population of newborns at the well-baby clinic with 0.09/1000 live births. Retrocochlear hearing loss is most common in children with risk factors for hearing disorders such as hyperbilirubinemia. Therefore, in contrast to the recommendation of using TEOAE-TEOAE for well-babies, newborns with known risk factors should always be screened using AABR-AABR.

To our knowledge, this is the first systematic review of quality measures using a two-stage screening design and quantitative data from original studies to assess the optimal screening algorithm. Strengths of the study include the large number of newborns included and the high number of study reports.

Because of the strict inclusion criteria (first inpatient screening, second screening within the first month), we did not include studies with outpatient screening only or screening within 6 weeks after birth. We chose these criteria in order to achieve the greatest possible homogeneity among the studies and to be more confident that the differences we found were due to the chosen screening algorithm rather than to differences in study setting or patient age. However, even with these strict inclusion criteria, the results showed a moderate amount of heterogeneity, so we lowered the overall level of evidence for this study.

One limitation of our study is the assumption of homogeneity of sensitivities and specificities across all studies, which ignores the heterogeneity, caused by differently qualified staff and in different settings (i.e., quiet vs. noisy). As these factors influencing the screening result are only described in detail in a few reports, they could not be considered in the meta-analysis. Similarly, the reports often lack information on whether data from children with risk factors for hearing impairment were included. However, they usually provide information on whether children from the NICU were included. In the subgroup analysis including only well babies, the AABR-AABR and TEOAE-TEOAE algorithms showed the lowest RFR (below 4%), and higher RFR were found after changing the test method. These findings confirm the overall results as the sampled population is less heterogeneous (only well babies).

A further limitation of our study was the inability to investigate the sensitivity of the different algorithms, as data on the outcome of babies with positive screening results were not available in the vast majority of studies. The implementation of standardized recording of children with hearing disorders, including the etiology (congenital or acquired), is necessary to enable the investigation of false-negative screening results and thus the sensitivity of the different algorithms.

In its most recent 2019 position paper, the Joint Committee on Infant Hearing recommends performing at least two screening attempts with the same method or an AABR after TEOAE before discharge of a well baby. TEOAE testing after an initial AABR with a refer result is also acceptable for well babies, as the lost-to-follow-up rate for outpatient follow-up is very high (6, 34). The UK screening program guidelines recommend performing a further TEOAE test with an interval of at least 5 h if the first TEOAE test is not passed for well babies (15).

The results of the meta-analysis and the data analysis of the German follow-up evaluation should provide evidence for adjusting the German Pediatrics Directive regarding the method of the second test to improve the RFR and align with international recommendations. Staff compliance with performing a second test before discharge is expected to improve if TEOAE tests are allowed, as TEOAE tests are faster and easier to perform than an AABR measurement. In contrast, changing the method of the second hearing test after failing the initial test results in a higher RFR without evident advantages. Therefore, the TEOAE-TEOAE screening algorithm for well babies could also be recommended for other countries, given that TEOAE represents a screening method that is reliable, cost-effective, and easy to apply in the clinical setting. However, in children with risk factors for perinatal hearing impairment, both hearing tests should always be performed with an AABR test, as specified in the German Pediatrics Directive (14) and international guidelines.

## Data Availability

Publicly available datasets were analyzed in this study. This data can be found at: Open Science Framework repository at https://osf.io/nuk4p/.
