# Supplementary material for: Quality measures of two-stage newborn hearing screening: systematic review and meta-analysis
Source: Front Public Health. 2025 Apr 16;13:1566478. doi: 10.3389/fpubh.2025.1566478 (PMC12041219; doi:10.3389/fpubh.2025.1566478)
Supplement: Supplementary file 1 [file Table_1.pdf]

Supplementary Material

1 Supplementary Data

1.1 Supplementary Data 1

Model

The study population consists of newborns with relevant hearing impairment (“diseased”, D^+) and those without (“healthy”, D^-). The prevalence of hearing impairment in the population is denoted by π. The first stage of screening is performed using a method with a sensitivity of SE1 and specificity of SP1. Therefore, the test positivity rate of the first stage (PR1), which includes true positives (TP1) and false positives (FP1), is given by: PR1 = TP1 + FP1 = π*SE1 + (1-π)*(1-SP1). These newborns are considered “*failed”* and should receive a second test. The rate of positive results from the first stage is also referred to as the “failure rate”.

The proportion of newborns whose first test result was “pass” and who therefore leave the screening process is the negative rate NR1 = π*(1-SE1) + (1-π)*SP1. However, not all newborns with a positive first test proceed to the second stage: The proportion ρ of positively tested newborns is *lost*. This loss is assumed to be independent of hearing status (newborns drop out for reasons unrelated to the first test result). Thus, a proportion of (1-ρ) newborns undergo the second stage screening test.

The proportion of SE2 is identified as positive among the true positives of the first test. Accounting for the loss, this yields TP2 = TP1*(1-ρ)*SE2 = π*SE1*(1-ρ)*SE2. Similarly, the proportion of (1-SP2) is falsely identified as positive among the false positives of the first test. Accounting for the loss, we obtain FP2 = FP1*(1-ρ)*(1-SP2) = (1-π)*(1-SP1)*(1-ρ)*(1-SP2).

The proportion of newborns with “fail” results in both tests, together with those who are lost after a first positive test, form the refer rate (RFR):

RFR = ρ*[π*SE1 + (1-π)*(1-SP1)] + (1-ρ)*[π*SE1*SE2 + (1-π)*(1-SP1)*(1-SP2)]. (Eq. 1)

The structure of the two-stage model, together with the respective selection and loss to follow-up processes is shown in Supplementary Figure 1.

Theoretical consideration of factors influencing the refer rate

We consider the relationship between the first stage positive rate (PR1 = π*SE1 + (1-π)*(1-SP1)) and the refer rate (RFR).

RFR = ρ*[π*SE1 + (1-π)*(1-SP1)] + (1-ρ)*[π*SE1*SE2 + (1-π)*(1-SP1)*(1-SP2)] =
ρ*PR1 + (1-ρ)*[π*SE1*SE2 + (1-π)*(1-SP1)*(1-SP2)] =

PR1 * {ρ + (1-ρ) * (π*SE1*SE2 + (1-π)*(1-SP1)*(1-SP2)) / (π*SE1 + (1-π)*(1-SP1))} =

PR1* {ρ + (1-ρ)*P(T2+|T1+) }. (Eq. 2)

In case of a small prevalence π (close to 0, inserting π=0 in Eq.2), it holds

$$RFR = PR1 * \{\rho + (1-\rho)*(1-SP2)\}. \quad \text{(Eq. 3)}$$

For small prevalence estimates, the RFR is linearly related to the failure (positive) rate of the first stage PR1. This linear relationship is determined by the loss rate ρ and the specificity of the second stage test SP2. Such a linear relationship between the RFR and failure rate can be exploited in a meta-regression. The quantity P(T2+|T1+) is called the conditional stage 2 positive rate (cPR2).

Equations 2 and 3 split the RFR into a component that quantifies the influence of the first stage and a component that quantifies the influence of the procedures after stage 1 (selection to stage 2 and quality of the second test). The RFR is the product of these two factors. It is of interest to quantify influence after stage 1.

2 Supplementary Figures

2.1 Supplementary Figure 1

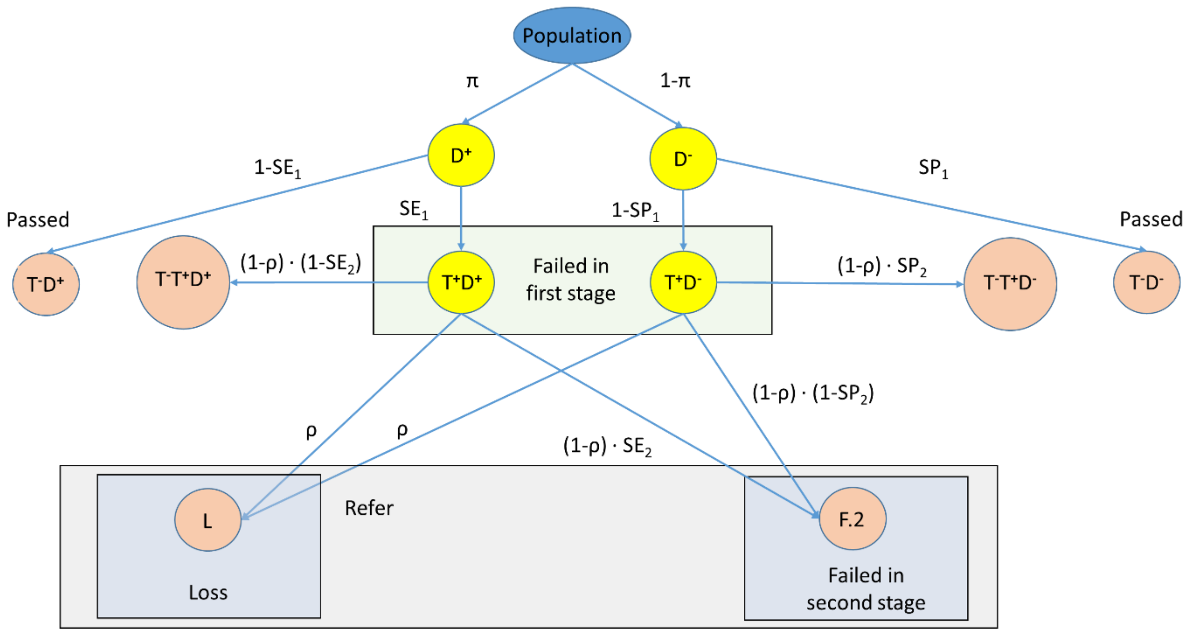

Supplementary Figure 1. Description of the model for two-stage newborn hearing screening. π = prevalence of hearing impairment, D+ (D-) = newborns with (without) hearing impairment, T- (T+) =newborns with "pass" ("fail") test results, SE_1 (SE_2) = sensitivity of the first (second) test, SP_1 (SP_2) = specificity of the first (second) test, ρ = loss rate after the first test stage, L = number of newborns lost after the first test result was "fail", F.2=number of newborns whose second test result was "fail".

2.2 Supplementary Figure 2

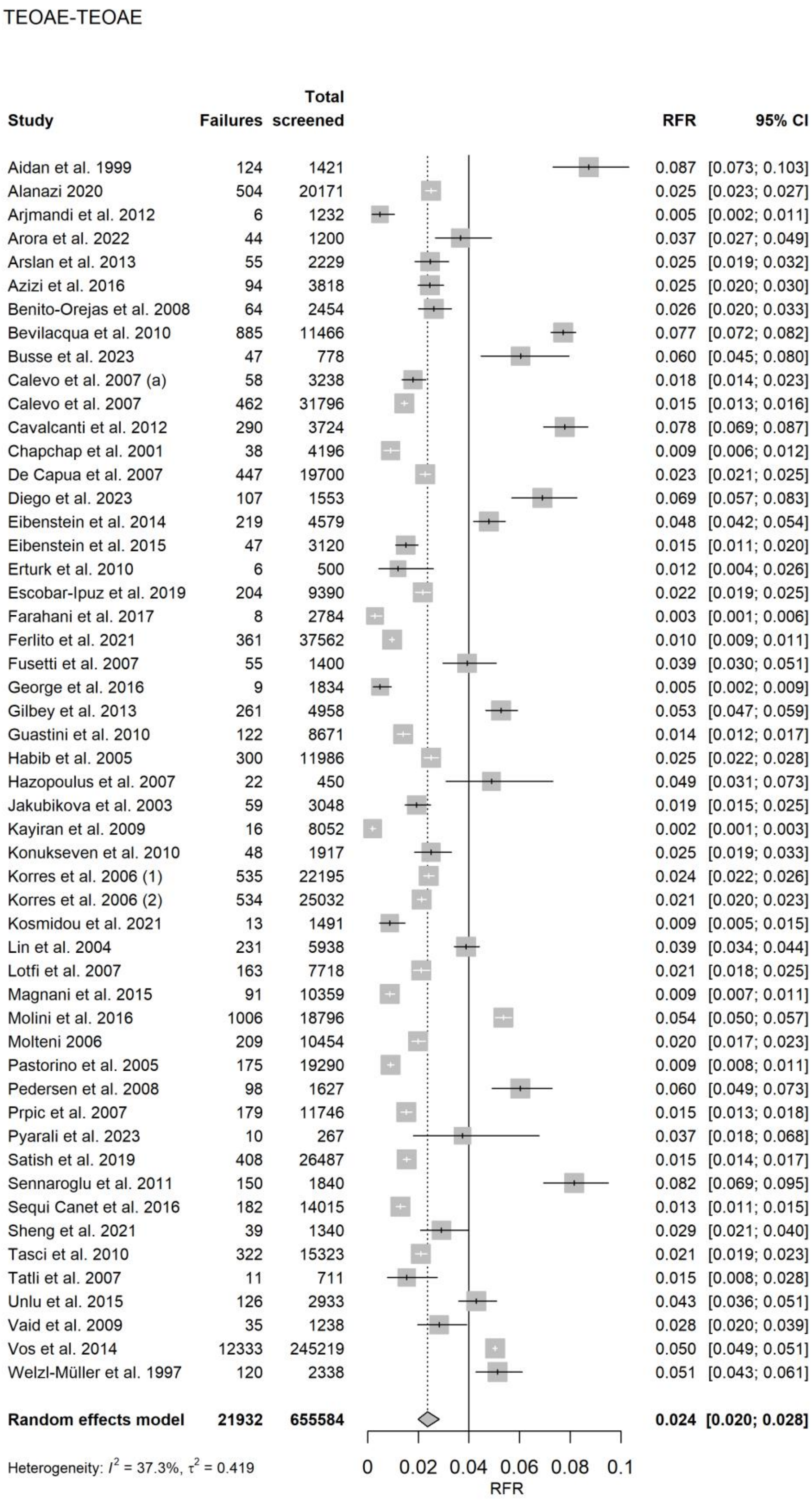

Supplementary Figure 2. Random-effects meta-analysis of refer rate (RFR) for 52 TEOAE-TEOAE study protocols without outliers. Excluding the studies by Clarke et al. 2003, Gül et al. 2013 and Yorulmaz et al. 2017. Shown are the number of newborns who did not pass first and second test (“Failures”), the number of screened newborns (“Total screened”), and the RFR with 95% confidence interval (95% CI) for each study. The summary estimate including the 95% CI is shown as a grey diamond on a scale ranging from 0% to 10%. The vertical solid line indicates the 4% threshold quality criteria defined in the Pediatrics Directive for the RFR. TEOAE = transient evoked otoacoustic emission.

2.3 Supplementary Figure 3

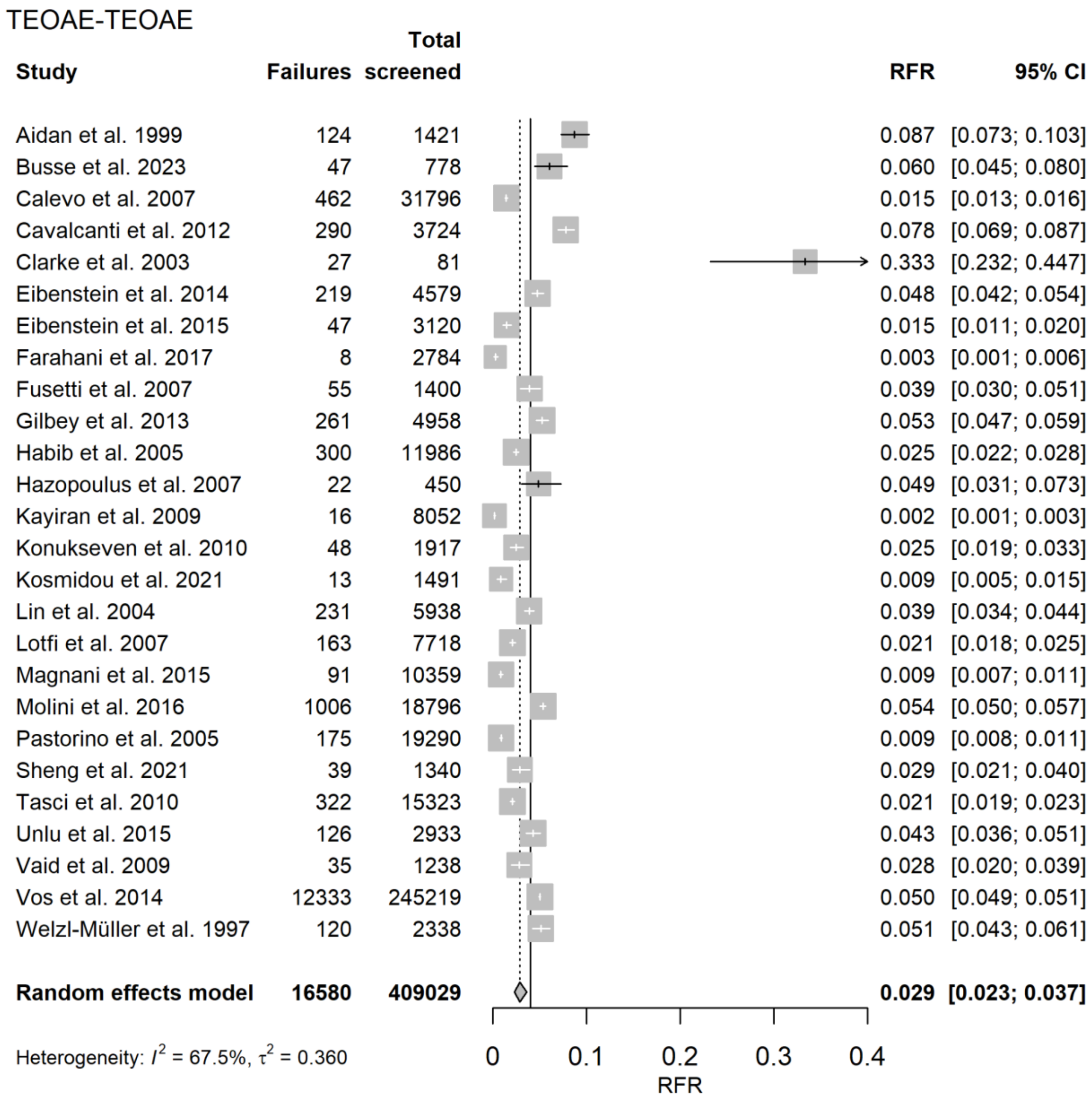

Supplementary Figure 3. Random-effects meta-analysis of refer rate (RFR) for the 26 TEOAE-TEOAE well-baby study protocols. Shown are the number of newborns who failed both the first and second test (“Failures”), the number of screened newborns (“Total screened”), and the RFR with the 95% confidence interval (95% CI) for each study. The summary estimate including the 95% CI is shown as a gray diamond on a scale ranging from 0% to 40%. The vertical solid line indicates the 4% threshold quality criteria defined in the Pediatrics Directive for the RFR. TEOAE = transient evoked otoacoustic emission.

2.4 Supplementary Figure 4

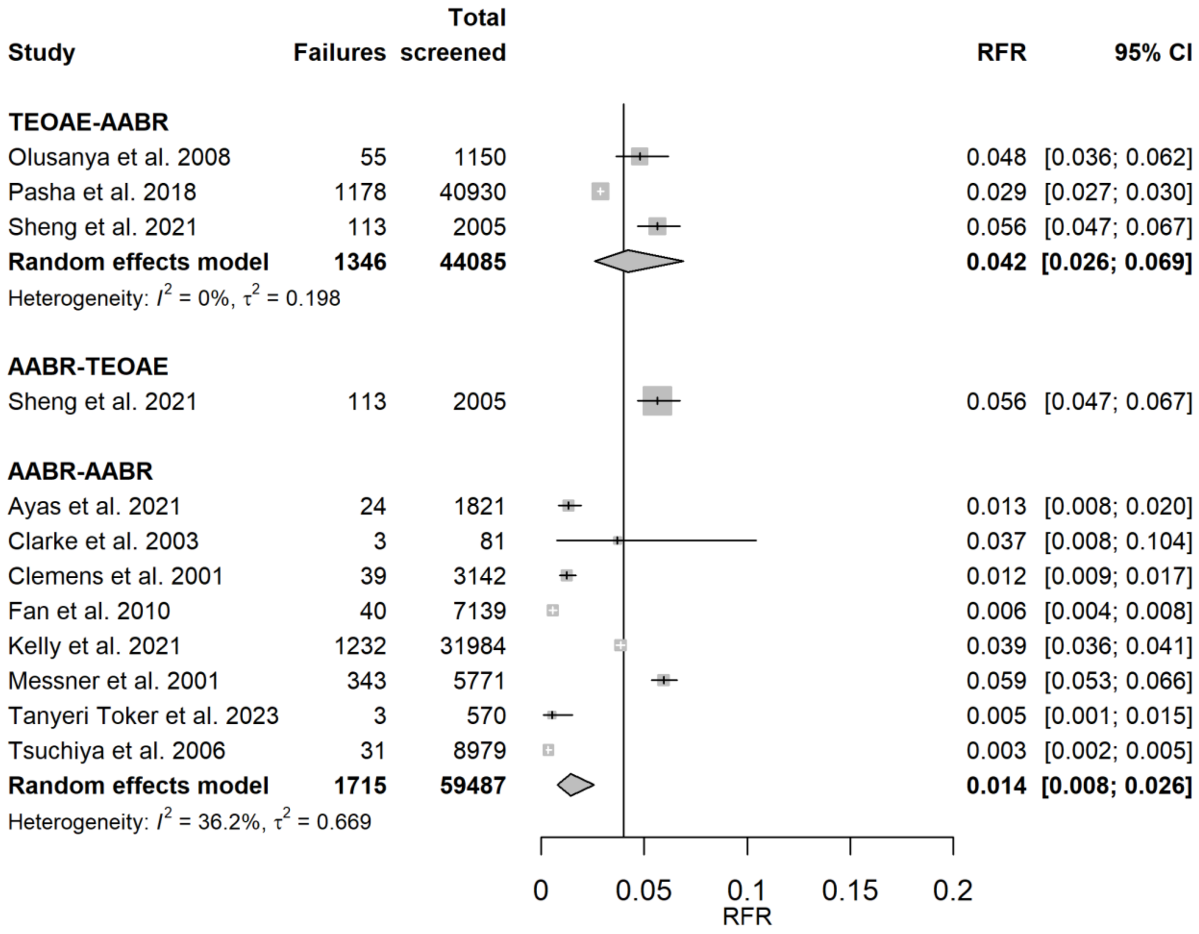

Supplementary Figure 4. Random-effects meta-analysis of refer rate (RFR) for TEOAE-AABR, AABR-TEOAE and AABR-AABR well-baby study protocols. Shown are the number of newborns who did not pass first and second test (“Failures”), the number of screened newborns (“Total screened”), and the RFR with the 95% confidence interval (95% CI) for each study. The summary estimate per test combination including the 95% CI is shown as a gray diamond on a scale ranging from 0% to 20%. The vertical solid line indicates the 4% threshold quality criteria defined in the Pediatrics Directive for the RFR. AABR = automated auditory brainstem response, TEOAE = transient evoked otoacoustic emission.

2.5 Supplementary Figure 5

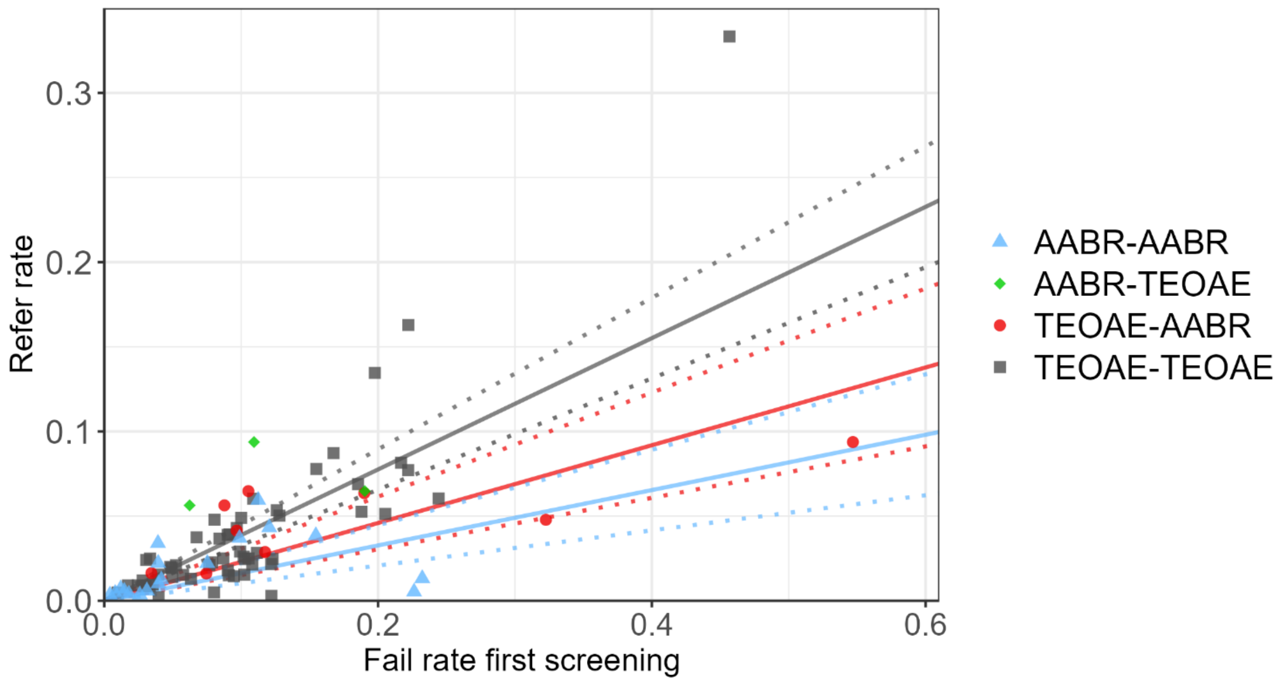

Supplementary Figure 5. Refer rates in dependence of the failure rate of the first test for the test combinations AABR-AABR (n=18, blue triangles), AABR-TEOAE (n=3, green diamonds), TEOAE-AABR (n=9, red dots) and TEOAE-TEOAE (n=55, gray squares). AABR = automated auditory brainstem response, TEOAE = transient evoked otoacoustic emission. Meta-regression lines including confidence intervals are plotted with data from Supplementary Table 1. No meta-regression line is shown for the AABR-TEOAE test combination with only three studies.

3 Supplementary Tables

3.1 Supplementary Table 1

Supplementary Table 1. Results of the meta-regression. SD = standard deviation, CI = confidence interval, TEOAE = transient evoked otoacoustic emission, AABR = automated auditory brainstem response.

Test combination	Mean slope (95% CI)
TEOAE-TEOAE	0.388 (0.328, 0.447)
TEOAE-AABR	0.230 (0.152, 0.307)
AABR-AABR	0.163 (0.104, 0.223)

3.2 Supplementary Table 2

Supplementary Table 2. Study-level assessment of bias for all 85 study protocols. TEOAE = transient evoked otoacoustic emission, AABR = automated auditory brainstem response.

	Risk of bias				Applicability concerns		
**Study**	**Patient selection**	**First test**	**Second test**	**Flow and timing**	**Patient selection**	**First test**	**Second test**
**TEOAE-TEOAE:**							
Aidan et al. 1999	low	low	low	high	high	low	low
Alanazi 2020	low	low	low	high	high	low	low
Arjmandi et al. 2012	high	low	low	high	high	Low	low
Arora et al. 2022	high	low	low	high	high	low	low
Arslan et al. 2013	high	low	low	low	high	low	low
Azizi et al. 2016	high	low	low	high	high	low	low
Benito-Orejas et al. 2008	high	low	low	high	high	low	low
Bevilacqua et al. 2021	low	low	low	high	high	low	low
Busse et al. 2023	low	low	low	high	high	low	low
Calevo et al. 2007 (a)	unclear	low	low	low	low	low	low

	Risk of bias				Applicability concerns		
**Study**	**Patient selection**	**First test**	**Second test**	**Flow and timing**	**Patient selection**	**First test**	**Second test**
Calevo et al. 2007	low	low	low	low	low	low	low
Calvacanti et al. 2012	high	low	low	high	high	low	low
Chapchap et al. 2001	high	low	low	high	high	low	low
Clarke et al. 2003	high	low	low	low	low	low	low
De Capua et al. 2007	high	low	low	high	high	low	low
Diego et al. 2023	high	low	low	high	high	low	low
Eibenstein et al. 2014	low	low	low	high	high	low	low
Eibenstein et al al 2015	low	low	low	high	low	low	low
Erturk et al. 2010	unclear	low	low	high	high	low	low
Escobar-Ipuz et al. 2019	high	low	low	low	high	low	low
Farahani et al. 2017	low	low	low	low	low	low	low
Ferlito et al. 2021	high	low	low	high	high	low	low
George et al. 2016	high	low	low	high	high	low	low
Fusetti et al. 2007	low	low	low	high	high	low	low
Gilbey et al. 2013	low	low	low	low	low	low	low
Guastini et al. 2010	high	low	low	low	high	low	low
Gül et al. 2013	high	low	low	high	high	low	low
Habib et al. 2005	low	low	low	low	low	low	low
Hatzopoulus et al. 2007	low	low	low	high	high	low	low
Jakubikova et al. 2003	high	low	low	high	high	low	low
Kayiran et al. 2009	high	low	low	low	high	low	low
Konukseven et al. 2010	low	low	low	high	high	low	low
Korres et al. 2006 (1)	high	high	low	high	high	high	low
Korres et al. 2006 (2)	low	low	low	low	low	low	low
Kosmidou et al. 2021	low	low	low	low	low	low	low
Lin et al. 2004	high	low	low	high	high	low	low
Lotfi et al. 2007	low	low	low	high	low	low	low
Magnani et al. 2015	low	low	low	low	low	low	low
Molini et al. 2016	low	low	low	high	high	low	low
Molteni 2006	high	low	low	high	high	low	low
Pastorino et al. 2005	low	low	low	high	high	high	low
Pedersen et al. 2008	high	low	low	high	high	low	low

	**Risk of bias**				**Applicability concerns**		
**Study**	**Patient selection**	**First test**	**Second test**	**Flow and timing**	**Patient selection**	**First test**	**Second test**
Prpic et al. 2007	high	low	low	low	high	low	low
Pyarali et al. 2023	high	low	low	low	high	low	low
Satish et al. 2019	high	low	low	low	high	low	low
Sennaroglu et al. 2011	high	low	low	unclear	high	low	low
Sequi Canet et al. 2016	low	low	low	high	high	low	low
Sheng et al. 2021	low	low	low	high	high	low	low
Tasci et al. 2010	low	low	low	low	high	low	low
Tatli et al. 2007	high	low	low	high	high	low	low
Unlu et al. 2015	low	low	low	low	low	low	low
Vaid et al. 2009	low	low	low	high	high	low	low
Vos et al. 2014	low	low	low	high	high	low	low
Welzl-Müller et al. 1997	low	low	low	high	high	low	low
Yorulmaz et al. 2017	low	low	low	high	high	low	low
**TEOAE-AABR:**							
Dort et al. 2000	high	low	low	low	high	low	low
Lin et al. 2007	low	low	low	low	low	low	low
Mazlan et al. 2022	low	low	low	high	high	low	low
Nennstiel-Ratzel et al. 2007	high	low	low	high	high	low	low
Olusanya et al. 2008	low	low	low	low	low	low	low
Olusanya et al. 2010	high	low	low	high	high	low	low
Ong et al. 2020	high	low	low	low	high	low	low
Pasha et al. 2018	low	low	low	high	high	low	low
Sheng et al. 2021	low	low	low	low	low	low	low
**AABR-TEOAE:**							
Dort et al. 2000	high	low	low	low	high	low	low
Ong et al. 2020	low	low	low	low	high	low	low
Sheng et al. 2021	low	low	low	low	low	low	low
**AABR-AABR:**							
Alothman et al. 2024	high	low	low	low	high	low	low
Al Shamisi et al. 2023	high	low	low	high	high	low	low
Ayas et al. 2021	low	low	low	low	low	low	low

	Risk of bias				Applicability concerns		
**Study**	**Patient selection**	**First test**	**Second test**	**Flow and timing**	**Patient selection**	**First test**	**Second test**
Benito-Orejas et al. 2008	low	low	low	high	high	low	low
Busse et al. 2023	high	low	low	low	high	low	low
Clarke et al. 2003	high	low	low	low	low	low	low
Clemens et al. 2001	low	low	low	low	low	low	low
Erturk et al. 2010	high	low	low	high	high	low	low
Fan et al. 2010	high	low	low	low	high	low	low
Gupta et al. 2015	high	low	low	low	high	low	low
Huang et al. 2013	high	low	low	low	high	low	low
Iwasaki et al. 2003	low	low	low	low	low	low	low
Kelly et al. 2021	low	low	low	low	low	low	low
Messner et al. 2001	low	low	low	high	high	low	low
Oruc et al. 2021	high	low	low	low	high	low	low
Shim et al. 2021	high	low	low	low	high	low	low
Tanyeri Toker et al. 2023	low	low	low	low	low	low	low
Tsuchiya et al. 2006	high	low	low	high	low	low	low

3.3 Supplementary Table 3

Population: (Well) babies undergoing a two-stage hearing screening

Setting: Newborns from studies from all countries (high, middle and low-income countries) with the following criteria

(1) Initial screening as an inpatient in the maternity clinic

(2) Second test up to a maximum of one month later

(3) No use of distortion product otoacoustic emissions (DPOAE)

(4) Not exclusively NICU (neonatal intensive care unit).

Includes studies of well babies only and studies of well babies and NICU infants. Studies of exclusively NICU infants were not included in this review.

Intervention: Two-stage hearing screening using TEOAE, AABR, or combination of both

Comparison: not applicable

Supplementary Table 3: Summary of findings table. CI = confidence interval, GRADE = GRADE Working Group grades of evidence (high, moderate, low, very low).[1]Level of evidence was lowered by one level due to found moderate heterogeneity in random-effects meta-analysis. [2]Level of evidence was lowered by one level due to small number of pooled studies in random-effects meta-analysis.

Test findings			Certainty of the evidence (GRADE)	Comments
**Screening algorithm**	**Pooled refer rate (95% CI)**	**Number of newborns (studies)**		
AABR-AABR	1.3% (0.9, 1.8%)	331526 (18)	Moderate[1]	Moderate heterogeneity, but larger number of studies
TEOAE-TEOAE	2.7% (2.2, 3.2%)	671721 (55)	Moderate[1]	Moderate heterogeneity, but large number of studies
TEOAE-AABR	3.9% (2.9, 5.1%)	120054 (9)	Moderate[2]	Moderate heterogeneity and small number of studies
AABR-TEOAE	5.9% (5.0, 6.9%)	2316 (3)	Moderate[2]	No evidence of heterogeneity, but small number of studies

3.4 Supplementary Table 4

Supplementary Table 4. PRISMA checklist. NA = not applicable.

Section and Topic	Item #	Checklist item	Location where item is reported
**TITLE**			
Title	1	Identify the report as a systematic review.	Title
**ABSTRACT**			
Abstract	2	See the PRISMA 2020 for Abstracts checklist.	Considered in abstract
**INTRODUCTION**			
Rationale	3	Describe the rationale for the review in the context of existing knowledge.	Introduction paragraphs 1-4
Objectives	4	Provide an explicit statement of the objective(s) or question(s) the review addresses.	Introduction paragraph 4
**METHODS**			
Eligibility	5	Specify the inclusion and exclusion criteria for the	Materials and methods

Section and Topic	Item #	Checklist item	Location where item is reported
criteria		review and how studies were grouped for the syntheses.	→ Eligibility criteria
Information sources	6	Specify all databases, registers, websites, organisations, reference lists and other sources searched or consulted to identify studies. Specify the date when each source was last searched or consulted.	Materials and methods → Search method
Search strategy	7	Present the full search strategies for all databases, registers and websites, including any filters and limits used.	Materials and methods → Search method
Selection process	8	Specify the methods used to decide whether a study met the inclusion criteria of the review, including how many reviewers screened each record and each report retrieved, whether they worked independently, and if applicable, details of automation tools used in the process.	Materials and methods → Study records
Data collection process	9	Specify the methods used to collect data from reports, including how many reviewers collected data from each report, whether they worked independently, any processes for obtaining or confirming data from study investigators, and if applicable, details of automation tools used in the process.	Materials and methods → Data extraction and items
Data items	10a	List and define all outcomes for which data were sought. Specify whether all results that were compatible with each outcome domain in each study were sought (e.g. for all measures, time points, analyses), and if not, the methods used to decide which results to collect.	Materials and methods → Data extraction and items
	10b	List and define all other variables for which data were sought (e.g. participant and intervention characteristics, funding sources). Describe any assumptions made about any missing or unclear information.	Materials and methods → Data extraction and items, legend of Table 1
Study risk of bias assessment	11	Specify the methods used to assess risk of bias in the included studies, including details of the tool(s) used, how many reviewers assessed each study and whether they worked independently, and if applicable, details of automation tools used in the process.	Materials and methods → Risk of bias in individual studies and publication bias
Effect	12	Specify for each outcome the effect measure(s) (e.g.	Materials and methods

Section and Topic	Item #	Checklist item	Location where item is reported
measures		risk ratio, mean difference) used in the synthesis or presentation of results.	→ Data extraction and items
Synthesis methods	13a	Describe the processes used to decide which studies were eligible for each synthesis (e.g. tabulating the study intervention characteristics and comparing against the planned groups for each synthesis (item #5)).	Materials and methods → Statistical analysis
	13b	Describe any methods required to prepare the data for presentation or synthesis, such as handling of missing summary statistics, or data conversions.	Materials and methods → Data extraction and items
	13c	Describe any methods used to tabulate or visually display results of individual studies and syntheses.	Materials and methods → Statistical analysis
	13d	Describe any methods used to synthesize results and provide a rationale for the choice(s). If meta-analysis was performed, describe the model(s), method(s) to identify the presence and extent of statistical heterogeneity, and software package(s) used.	Materials and methods → Statistical analysis
	13e	Describe any methods used to explore possible causes of heterogeneity among study results (e.g. subgroup analysis, meta-regression).	Materials and methods → Subgroup analysis
	13f	Describe any sensitivity analyses conducted to assess robustness of the synthesized results.	Materials and methods → Subgroup analysis
Reporting bias assessment	14	Describe any methods used to assess risk of bias due to missing results in a synthesis (arising from reporting biases).	Materials and methods → Risk of bias in individual studies and publication bias
Certainty assessment	15	Describe any methods used to assess certainty (or confidence) in the body of evidence for an outcome.	Materials and methods → Confidence in cumulative evidence
**RESULTS**			
Study selection	16a	Describe the results of the search and selection process, from the number of records identified in the search to the number of studies included in the review, ideally using a flow diagram.	Fig 1
	16b	Cite studies that might appear to meet the inclusion criteria, but which were excluded, and explain why they were excluded.	Legend of Fig 1
Study	17	Cite each included study and present its	Table 1

Section and Topic	Item #	Checklist item	Location where item is reported
characteristics		characteristics.	
Risk of bias in studies	18	Present assessments of risk of bias for each included study.	S2 Table
Results of individual studies	19	For all outcomes, present, for each study: (a) summary statistics for each group (where appropriate) and (b) an effect estimate and its precision (e.g. confidence/credible interval), ideally using structured tables or plots.	Fig 2
Results of syntheses	20a	For each synthesis, briefly summarise the characteristics and risk of bias among contributing studies.	Fig 3
	20b	Present results of all statistical syntheses conducted. If meta-analysis was done, present for each the summary estimate and its precision (e.g. confidence/credible interval) and measures of statistical heterogeneity. If comparing groups, describe the direction of the effect.	See Figs 3 and 4 and Tables 2 and 3
	20c	Present results of all investigations of possible causes of heterogeneity among study results.	S2 Fig – S4 Fig
	20d	Present results of all sensitivity analyses conducted to assess the robustness of the synthesized results.	S2 Fig - S4 Fig
Reporting biases	21	Present assessments of risk of bias due to missing results (arising from reporting biases) for each synthesis assessed.	NA, since no reporting bias occurred.
Certainty of evidence	22	Present assessments of certainty (or confidence) in the body of evidence for each outcome assessed.	S3 Table
**DISCUSSION**			
Discussion	23a	Provide a general interpretation of the results in the context of other evidence.	Discussion paragraphs 3-5
	23b	Discuss any limitations of the evidence included in the review.	Discussion paragraph 6
	23c	Discuss any limitations of the review processes used.	Discussion paragraph 7
	23d	Discuss implications of the results for practice, policy, and future research.	Discussion last paragraph
**OTHER INFORMATION**			
Registration and protocol	24a	Provide registration information for the review, including register name and registration number, or state that the review was not registered.	Materials and methods → Study protocol

Section and Topic	Item #	Checklist item	Location where item is reported
	24b	Indicate where the review protocol can be accessed, or state that a protocol was not prepared.	Materials and methods → Study protocol
	24c	Describe and explain any amendments to information provided at registration or in the protocol.	Materials and methods → Study protocol
Support	25	Describe sources of financial or non-financial support for the review, and the role of the funders or sponsors in the review.	See Additional information
Competing interests	26	Declare any competing interests of review authors.	See Additional information
Availability of data, code and other materials	27	Report which of the following are publicly available and where they can be found: template data collection forms; data extracted from included studies; data used for all analyses; analytic code; any other materials used in the review.	Materials and Methods → Statistical analysis
